# Audiologists’ attitudes and practice toward referring for psychosocial intervention with cochlear implant patients

**DOI:** 10.3389/fresc.2023.1306485

**Published:** 2024-01-04

**Authors:** Sarah E. Warren, Autumn L. Barron

**Affiliations:** Department of Communication Sciences and Disorders, University of Memphis, Memphis, TN, United States

**Keywords:** person-centered care, audiology, cochlear implant, communication sciences and disorders (CSD), psychosocial care, social work, psychology, counseling

## Abstract

**Background:**

Hearing loss is associated with a range of poor psychosocial outcomes. Cochlear implants (CI) are an available treatment option for significant hearing loss and have been linked to improved quality of life in patients. Evidence suggests that audiologists lack the skills to appropriately detect, address, and refer for psychosocial needs among patients with hearing loss. The objective of this study is to examine the attitudes and practice patterns related to psychosocial care among audiologists who work with CI users.

**Methods:**

A cross-sectional survey was administered to clinical audiologists who work with CI recipients in the United States. The survey evaluated participants’ attitudes toward psychosocial services and factors that contribute to their abilities to address the psychosocial needs of their patients. Additionally, participants were surveyed about their practice patterns including the use of psychosocial screeners, clinical protocols regarding psychosocial care, and referral patterns for coordinated psychosocial services. Descriptive statistics were used to summarize survey responses.

**Results:**

Sixty-eight audiologists completed the survey. Of these audiologists, a majority (73.6%) held the attitude that most or all CI patients would benefit from psychosocial intervention. Despite clinicians’ recognition of psychosocial needs in this population, over 90% of participants reported never screening for psychosocial symptoms. Additionally, a majority of respondents indicated that they seldom refer their patients for psychosocial services, with referrals occurring less than half the time (58%) or never (27%). Additionally, few audiologists reported utilizing protocols or resources for guiding psychosocial practices. Audiologists indicated that the primary factors that influence their psychosocial practices include time available to spend with the patient and their comfort level in counseling.

**Conclusion:**

Audiologists working with CI patients recognize the potential benefit of psychosocial intervention in this population. Nevertheless, audiologists encounter barriers in clinical practice which limit their ability to identify and address the psychosocial needs of their patients. Strategies designed to enhance audiologists’ capacity to recognize the psychosocial needs of CI users, in addition to improved interprofessional practice on CI teams, implies significant opportunities to improve the provision of patient-centered hearing care.

## 1 Introduction

Psychosocial consequences of hearing loss are far-reaching and encompass various aspects of individuals’ lives. Research has shown that hearing loss is associated with social isolation and loneliness [e.g., (1–3)], stigma [e.g., (4, 5)], anxiety and depression [e.g., (6)], reduced family and community engagement [e.g., (7)], and psychological distress [e.g., (8)]. Negative psychosocial effects extend beyond the person with hearing loss. Communication partners (spouses, close family and friends, or caregivers) may experience negative psychosocial effects related to the disruptions in communication (9). Specifically, communication difficulties stemming from hearing loss are associated with increased caregiver burden (10, 11) and relationship distress (12). The burden of hearing loss has a greater impact on individuals with more severe degrees of hearing loss (13). Cochlear implants (CI) are an established treatment of communication disorders resulting from debilitating hearing loss for both children and adults. The psychosocial benefits of CI have been documented in individuals with hearing loss across the lifespan since their introduction three decades ago [e.g., (14–17)].

A shift in healthcare over the past two decades has increased emphasis on patient-centeredness, which is viewed favorably by audiologists (18). Patient-centered practice is associated with improved patient outcomes including patient satisfaction, patient adherence to recommendations (19, 20). In applying patient-centered practices, audiologists place focus on patients and their support system (family and caregivers) by integrating psychosocial counseling as part of their intervention and audiological rehabilitation (21). Recognizing the role of psychosocial factors is critical to patient-centered care, and counseling is considered a necessary provision in the field of audiology (22). Fundamental counseling skills are provided to audiologists as part of graduate-level educational training, particularly in the areas of emotional support and informational counseling (23, 24). However, counseling education in the field is variable in terms of teaching methods and student evaluation (23).

Despite graduate-level counseling training provided to audiologists, some patients and families may have psychosocial needs which require intervention that is best delivered by a trained mental health professional. Professionals which specialize in psychosocial services have high-level training in counseling assessment, diagnosis, and intervention for treating individuals with a range of mental health issues. This training surpasses the expectations of audiologists, and the provision of many specific psychosocial interventions (such as cognitive behavioral therapy and dialectical behavioral therapy) requires a masters or doctoral degree, postgraduate training, and in some cases, certification and/or licensure (25). An interdisciplinary team-based approach is an integral part of the aural rehabilitation process associated with cochlear implantation (26, 27). Collaboration with mental health professionals such as trained social workers and psychologists can enhance patient-centered care in this population.

The involvement of mental health professionals as part of the CI team can be traced back to the earliest literature on single channel devices (26) when the effectiveness of CI on psychosocial wellbeing following cochlear implantation was first reported (28, 29). Subsequently, comprehensive psychosocial evaluations prior to cochlear implantation were recommended as standard clinical practice to evaluate cognitive and emotional appropriateness for device recommendation (26, 29, 30), particularly in the time that cochlear implantation was still largely considered an experimental procedure. The additional benefits of these evaluations in providing post-surgical support are also noted (26). As the effectiveness of CI was established, the practice of routine psychosocial evaluation evolved into a suggestive approach and was eventually phased out of most CI protocols (26).

Accessing psychosocial services can be a complex process, requiring individuals to overcome barriers related to affordability, availability, and stigma (31). Individuals with hearing loss face greater barriers to accessing mental health care compared to the general population, which are linked to an underutilization of mental health services (32). Policy in the United States have attempted to address the underutilization of mental health care, by both The Mental Health Parity and Addiction Equity Act of 2008 and the Affordable Care Act of 2021, which both expanded benefits for mental health services (33, 34). Regardless, underutilization of mental health services persists among individuals with hearing loss. Because of this disparity, the role of audiologists in working with CI recipients and their families becomes crucial in addressing the broader psychosocial implications related to fostering improved communication and relationships for both the individual and their communication partners.

The field of audiology lacks well-defined standards for providing evidence-based psychosocial care within its practice (24, 25, 35). Professional organizations such as the American Speech-Language and Hearing Association (ASHA) and American Academy of Audiology (AAA) acknowledge the inclusion of emotional support within the scope of practice for audiologists (36, 37). These organizations specify that an audiologist's scope of practice includes educational counseling, instruction, and psychosocial adjustment to hearing loss and related interventions. Management of audiological conditions can be complex, presenting with a range of psychosocial conditions (such as anxiety and depression) and expanding upon an audiologist's skillset. These cases require detection, assessment, and intervention which most audiologists are not prepared to provide (25). The national standards provided by ASHA and AAA (38) do not provide specific guidance on assessing psychosocial health or referrals to mental health professionals (25, 36, 39). ASHA provides guidance on informational and personal adjustment counseling through their practice portal on their website (https://www.asha.org/practice-portal/professional-issues/counseling-for-professional-service-delivery/). Regardless, the threshold in which a patient or family's psychosocial intervention needs surpasses the audiologist's scope of practice is not clearly defined.

Research indicates that a majority of audiologists are not adequately detecting, addressing, and referring patients with poor psychosocial well-being (23, 40, 41), and do not consistently communicate in ways that align with patient-centered communication principles (42). Audiologists’ attitudes and practices toward psychosocial health have been investigated [e.g., (23, 40, 41, 43, 44)]; however, this has not been specifically explored among audiologists in relation to referral patterns while working with CI recipients and their families. This topic is of specific interest given the impact of severe-to-profound hearing loss on psychosocial function in addition to the historical context of psychosocial assessment and intervention in this population. The aims of this study were (1) to assess audiologists’ attitudes toward professional psychosocial intervention among CI recipients, and (2) to explore clinical practice patterns in relation to counseling and referring to mental health professionals (i.e., psychologist or social worker).

## 2 Material and methods

### 2.1 Design

This study was conducted as a cross-sectional survey. This survey was designed to evaluate attitudes and practice patterns related to psychosocial counseling and referral patterns among audiologists who work with CI recipients and their families. Participants completed an electronic survey where they provided both closed and open-ended responses.

### 2.2 Participants

Recruitment for this survey targeted certified clinical audiologists working with CIs in adult and pediatric populations in the United States. Participants were recruited between February and May 2022 through scripts posted to relevant social media groups, e-mail, and through the American Cochlear Implant Alliance listserv database.

A total of 80 individuals initiated this electronic survey, and 68 respondents completed the first section of the survey. The second section of the survey had an attrition of 8 participants, leaving the final participant count at 60 participants. A return rate could not be calculated due to online recruitment methods which did not capture how many potential participants declined to participate by not engaging in the survey. Prior to participation, individuals were informed that they would be asked about attitudes and practice patterns related to psychosocial referral patterns specific to CI recipients. Participants consented to the study by accepting the consent statement prior to engaging in the survey and were instructed to take the 15–20 min survey at a time and place of their preference. Approval for this study was obtained from the Institutional Review Board at the University of Memphis.

### 2.3 Instrument

The survey (see Supplementary Appendix) consisted of 25 items including multiple choice questions, Likert responses, and free-text responses. The survey was developed by the study investigators based on evidence regarding audiologists’ attitudes and referral practices toward professional psychosocial care while working with individuals who have hearing loss and utilize CI. The survey consisted of participant demographic and professional characteristics and of two content areas which included (1) audiologist attitudes toward referrals to professionals specializing in psychosocial services, and (2) practice patterns related to counseling and referring to professionals specializing in psychosocial services (defined as psychologists and social workers). Participants were allowed to move through this survey freely and at their own pace.

The first section of the survey collected clinician demographics including years of experience, gender, primary employment setting, region of practice (United States), number of graduate-level courses taken related psychosocial counseling, and clinical population in which the participant works. This section also collected information on individuals’ attitudes toward formal psychosocial services (specified as a psychologists or social worker) among CI recipients and their families.

In the second section of the survey, participants were provided a range of questions related to practice patterns. First, participants were provided with list of psychosocial screeners and responded to each assessment on a 3-point Likert scale indicating they administered this instrument “always”, “sometimes”, or “never.” Participants were also provided the opportunity to provide the name of any screener or assessment that they administered but was not included in the survey. Following these questions, participants were then provided with a list of potential factors which may influence a practitioner's counseling practices or decision to refer for psychosocial care. Participants responded to each potential factor on a 4-point Likert scale indicating if each factor affected their referral patters “a lot”, “a moderate amount”, “a little”, or “no impact.” Next, participants were provided with a list of potential referral situations and asked if they would refer to a psychosocial provider given each situation. The participants were given 3 discrete responses of “yes”, “no”, and “unsure.” The final questions in this section requested information regarding any official or unofficial clinic protocols related to psychosocial services. The survey concluded by giving individuals the opportunity to provide additional information about their psychosocial practices in an open-ended response.

### 2.4 Data analysis

Data were recorded in Qualtrics and was exported to a Microsoft Excel file. Quantitative analyses were performed using SPSS Statistical Software (Version 27.0.1, IBM Corporation). Frequency tables, mean values, and standard deviations were calculated descriptively. Qualitative, open-ended responses were documented in a Microsoft Excel file and analyzed for trends, resulting in the following information: (1) other professionals or individuals referred to for the purpose of psychosocial support, and (2) other notable approaches to psychosocial care in this population, which allowed individuals to share any perspectives which were not directly asked as part of the survey.

The authors were interested in a potential relationship between years of experience and practice replated to psychosocial care. Participants were assigned to one of three categories based on reported year of experience working with CI recipients. A chi-squared test of independence was performed to examine the relationship between professional experience with CI and responses multiple categories. A Fisher's Exact test was used in the case that categorical cell counts were less than 5. To estimate the number of participants needed for this evaluation, an *a priori* power analysis was conducted using G*Power version 3.1.9.7. The authors selected a medium effect size (.4), a significance criterion of *α* = .05, and power = .80. The minimum sample size needed was determined to be *n* = 61.

## 3 Results

### 3.1 Demographics and attitudes toward psychosocial care

Sixty-eight individuals completed this section of the survey. The participating audiologists had an average age of 10 years experiences as an audiologist and an average experience of 7 years of professional experience with CI. Of this participant pool, 24 audiologist (*n* = 24/65, 35%) reported 0–4 years’ experience, 24 audiologist (*n* = 24/65, 35%) reporting 4–11 years’ experience, and 20 audiologist (*n* = 20/68, 29%) reporting 10 or more years’ experience. Most surveyed audiologists (*n* = 64/68, 94%) were female, which generally represents the gender balance of the audiology profession in the United States (ASHA 2022 member and affiliate profile). When asked about their graduate-level educational preparation in counseling, ten audiologists (*n* = 10/68, 15%) indicated no formal course in counseling, thirty-five audiologists (*n* = 35/68; 51%) indicated one formal course in counseling, and twenty-three audiologists (*n* = 23/68; 34%) indicated two or more courses in counseling. Participant demographic information is outlined in Table 1.

**Table 1:** Study participant demographics (*N* = 68).

Years of clinical experience (year)	*N*	%
0–4	24	35.3%
5–11	24	35.3%
12+	20	29.4%
Sex		
Female	64	94.1%
Male	4	5.9%
Region (United States)		
Northeast	14	20.6%
Southeast	26	38.2%
Midwest	13	19.1%
West	15	22.1%
Practice setting		
Hospital	38	55.9%
University clinic	10	14.7%
ENT clinic	13	19.1%
Private practice	3	4.4%
Clinical population		
Children	13	19.1%
Adults	22	32.4%
Both	33	48.5%
Graduate-level courses taken in counseling		
0 courses	10	14.7%
1 course	35	51.5%
2 or more courses	23	19.1%

All participants indicated that they held the attitude that least some CI recipients and families would benefit from a referral to a professional specializing in psychosocial services as part of the CI rehabilitation process, with a majority of participants indicating that most (*n* = 28/68, 41%) or all (*n* = 22/68, 32%) CI patients or their families would benefit. A smaller proportion reported that only half (*n* = 14/68, 20%) or few (*n* = 4/68, 6%) would benefit from formal psychosocial intervention. Participants were then asked to estimate the benefit for the average patient or family who they would refer for professional psychosocial services. Participants reported that they held the attitude that their CI recipients would find it extremely beneficial (*n* = 10/68, 15%), very beneficial (*n* = 31/68, 46%) or probably beneficial (*n* = 27/68, 40%). Clinician attitudes toward professional psychosocial intervention as part of the CI rehabilitation process are outlined in Table 2.

**Table 2:** Participants were asked what percentage of patients would benefit from counseling with a psychosocial professional, and how effective the participant thinks the service would be to the average patient referred to professional psychosocial services. (*N* = 68).

What proportion would benefit	*N*	(%)
All patients	22	32.4%
Most patients	28	41.2%
About half of all patients	14	20.1%
A few patients	4	5.8%
No patients	0	0%
For the average patient you would refer, how effective would these services be?		
Extremely beneficial	10	14.7%
Very beneficial	31	45.6%
Probably beneficial	27	39.7%
Probably not beneficial	0	0%
Not at all beneficial	0	0%

### 3.2 Practice patterns related to psychosocial care

#### 3.2.1 Mental health screeners

**Figure 1:**
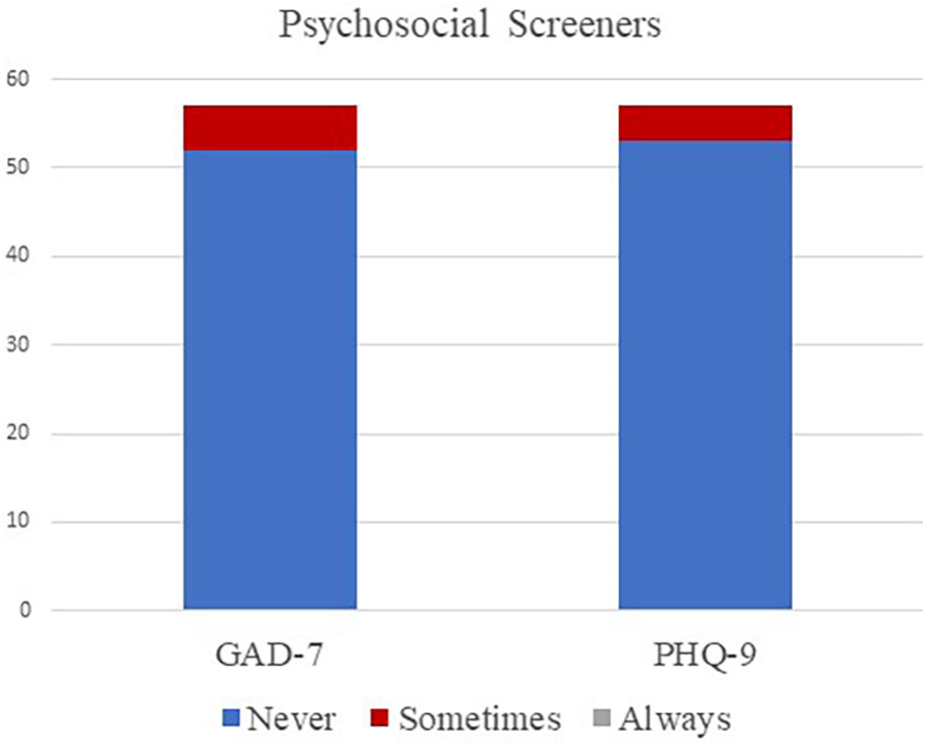
Participants’ responses to questions regarding psychosocial screening practices.

Sixty participants completed the second section of this survey, although not every participant answered every question. Participants were asked if they screened participants or families for anxiety using the 7-item Generalized Anxiety Disorder Scale [GAD-7; (45)] or 9-item Patient Health Questionnaire [PHQ-9; (46)]. Few participants (*n* = 6/57, 11%) reported using any screener at least some of the time. Specifically, 5 participants (*n* = 5/57, 9%) reported using the GAD-7 sometimes and 4 participants (*n* = 4/57, 7%) reported using the PHQ-9 sometimes. The majority of participants (*n* = 53/57, 93%) reported never using psychosocial screeners in their clinical practice. No other screeners related to screening psychosocial health were reportedly used. Results can be found in Figure 1.

#### 3.2.2 Referral indications related to psychosocial care

**Figure 2:**
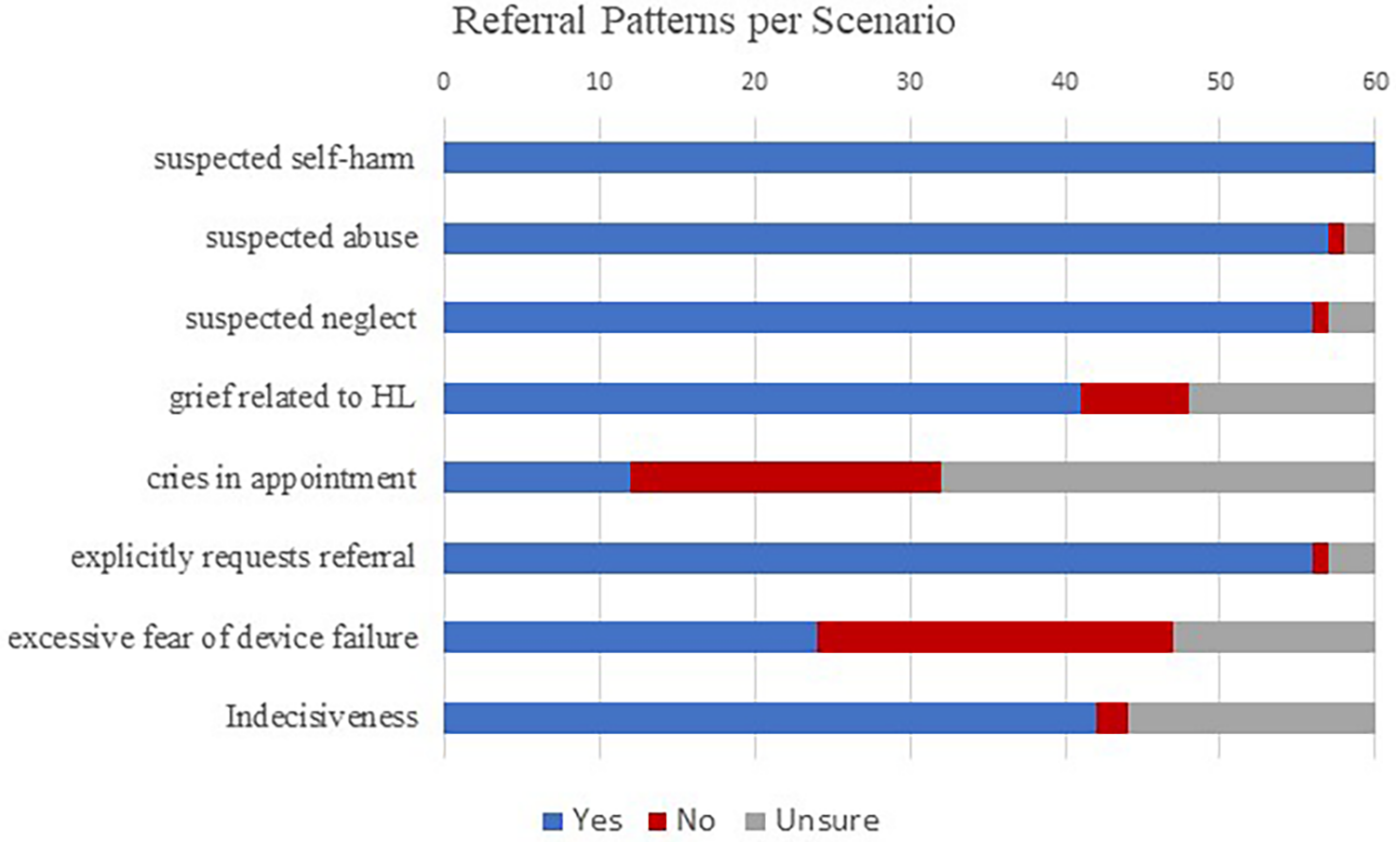
Participants responses to the question if they would generally refer patients given a range of clinical scenarios.

In general, the majority of participants reported that they would refer to a professional specializing in psychosocial services given a range of prospective situations. Participants were definitive in their decision to refer in cases of suspected self-harm, with all participants (*n* = 60/60, 100%) reporting that they would refer to psychosocial care. Participants generally agreed that patients should be referred for psychosocial intervention in the case of suspected neglect (*n* = 56/60, 93%) and abuse (*n* = 57/60, 95%), although a few participants reported being unsure about referring (*n* = 3/60, 5%; *n* = 2/60, 3% respectively) or would not refer (*n* = 1/60, 2%; *n* = 1/60, 2% respectively) in these cases. Likewise, most participants responded affirmatively that they would refer to psychosocial care if the patient or family explicitly requested a referral (*n* = 56/60, 93%), although some participants were unsure (*n* = 3/60, 5%) or unwilling (*n* = 1/60, 2%) to refer in that situation. Most participants indicated that they would refer in the situation of grief expression related to hearing loss (*n* = 41/60, 68%) and indecisiveness regarding the decision to pursue CI (*n* = 42/60, 70%). Participant responses were mixed regarding if they would refer (*n* = 24/60, 40%) or would not refer (*n* = 23/60, 38%) in cases involving fears related to device performance such as failure. See Figure 2 for responses related to referral patterns related to clinical situation.

**Figure 3:**
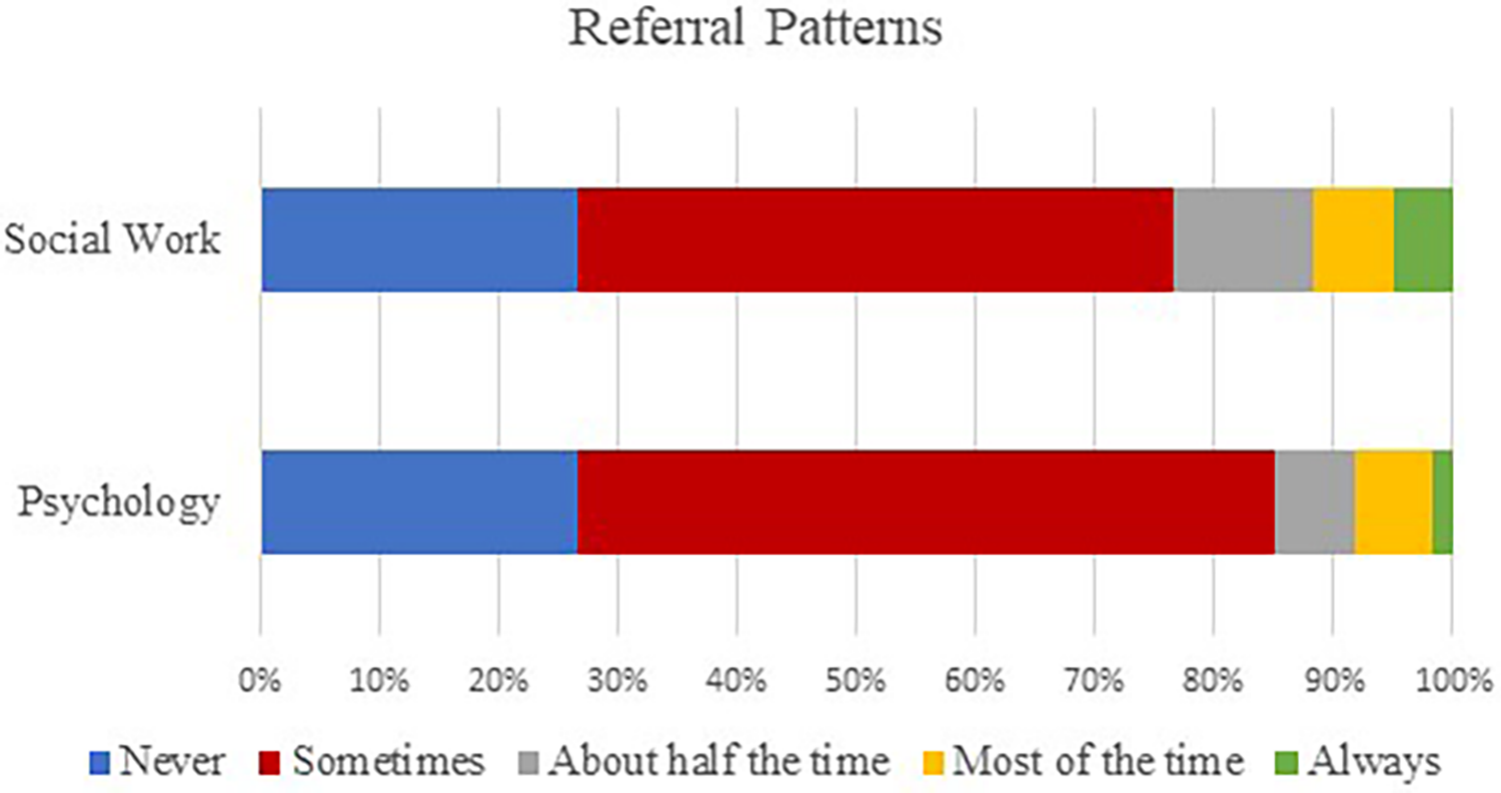
Participants’ frequency of referral to a social worker or psychologist.

Participants were asked to estimate how frequently they referred CI recipients or families to either psychology or social work. The most consistent response from participants was that they refer to these professionals occasionally/sometimes (range 30–35/60; 50%–58%). Social work had slightly more consistent referrals than psychology, with 23% of respondents (*n* = 14/60) reporting they refer to social work half the time or more compared to 15% of respondents (*n* = 9/60) reporting that they refer to psychology half the time or more. One participant (*n* = 1/60, 2%) reported referring to both psychology and social work for every patient/family, and two participants (*n* = 2/60, 3%) report referring to both psychology and social work for most patients/families. Nine participants (*n* = 9/60, 15%) reported never referring to psychology, social work, or other professional specializing in psychosocial services. See Figure 3 for results. Data were attempted to be analyzed to evaluate the relationship between years’ experience and practice patterns. Chi square testing was attempted, but cell counts were too small for reliable comparisons.

#### 3.2.3 Barriers to psychosocial intervention

**Figure 4:**
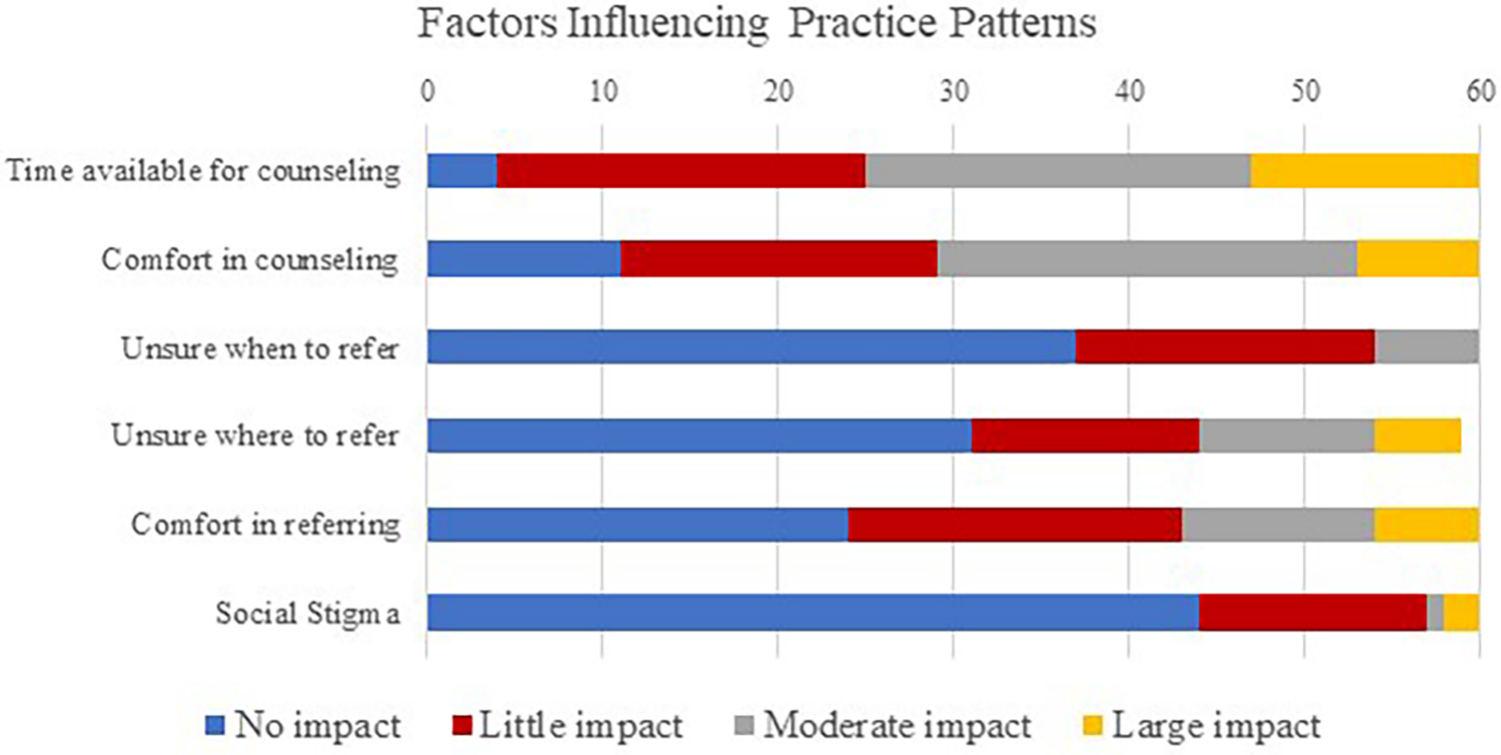
Participant responses to the influence of factors on practice patterns.

Participants were then asked to rate factors which influenced their counseling practice patterns using a 4-point Likert scale from “Impacts a lot” to “Does not impact at all”. The time available for counseling had the largest impacts on a participant's referral patterns, with 54 participants (*n* = 54/60, 90%) reporting time available for counseling had at least some impact on counseling patterns, and 35 participants (*n* = 35/60, 58%) reporting this had a moderate or great impact on counseling patterns. The participant's comfort level in counseling also influenced counseling patterns, with 49 participants (*n* = 49/60, 82%) reporting this factor having an impact. Knowing when and where to refer for psychosocial services was reported to have low influence practice patterns, with more than half participants reported no impact from these factors (*n* = 37/60, 62% and *n* = 31/59, 52% respectively). Finally, participants reported a range in confidence in making a referral to a professional who specialities in psychosocial services, with 6 participants (*n* = 6/60, 10%) reporting a large impact, 11 participants (*n* = 11/60, 18%) reporting a moderate impact, 19 participants (*n* = 19/60, 32%) reporting a small impact, and 24 participants (*n* = 24/60, 40%) reporting no impact. Stigma related to psychosocial care reportedly had low influence on practice patterns, as 44 participants (*n* = 44/60, 73%) reported social stigma having no influence over their practice. See Figure 4 for responses on factors of influence on counseling practice patterns and referrals to professionals who specialize in psychosocial services.

#### 3.2.4 Standards of practice

Participants were asked a rage of questions regarding their current clinical standards of practice. When asked if their clinic or CI team had a written protocol for referring for psychosocial services, 61 participants (*n* = 61/65, 94%) responded that they had no such protocol. Participants were then asked if they provide patients with a list of recommended professionals specializing in psychosocial care. In response to this question, 28 (*n* = 28/60, 47%) participants reported not having a recommendation list. Nineteen (*n* = 19/60, 32%) participants reported not having a formal list but providing patients with common suggestions. Thirteen participants (*n* = 13/60, 22%) reported having a recommendation list. Ten participants of (*n* = 10/60, 17%) indicated that this list was given out as needed and 3 participants (*n* = 3/60, 5%) indicating that the list was provided to all CI recipients or families. See Table 3 for a summary of these results.

**Table 3:** Participants responses regarding if they had a written protocol for psychosocial counseling, and if they had a formal list of referral resources or resources to provide to patients and families.

Written protocol (*N* = 65)	*N*	(%)
Yes	4	6.2%
No	61	93.8%
Referral resources (*N* = 60)		
A list of resources is provided to all patients/families	3	5.0%
A list of resources is provided to patients/families as needed	10	16.7%
No list of resources, but common suggestions are provided	19	31.7%
No resource or suggestions are provided	28	46.7%

After completing all questions, participants were encouraged to share additional information they deemed valuable related to psychosocial care of CI recipients and families. Fourteen participants (*n* = 14/60, 23%) reported they refer for psychosocial support to individuals other than psychologists and social workers. When allowed the opportunity to share about their referral practices in freeform text, the most common response was the use of peer mentors to support psychosocial wellbeing of CI recipients (*n* = 6/60; 10%). Other reported psychosocial support personnel included otolaryngologist (*n* = 3/60, 5%), behavioral medicine team (*n* = 3/60, 5%), school counselor (*n* = 3/60, 5%), vocational rehabilitation counselor (*n* = 1/60, 2%), pastoral care (*n* = 1/60, 2%), geriatric specialists (*n* = 1/60, 2%), support groups (*n* = 1/60, 2%), and “other healthcare providers” (*n* = 1/60, 2%).

## 4 Discussion

A survey of 68 participants explored the attitudes, counseling practices, and referral patterns of audiologists working in the United States in relation to professional psychosocial intervention as a part of routine clinical care among CI recipients and families. In summary, participants reported a positive attitude related to the benefits of psychosocial care among CI patients and their families and a willingness to refer to professional psychosocial care in a range of situations. However, when asked about routine practices related to identifying needs and referring to outside professionals, responses were inconsistent with attitudes towards psychosocial care. While these findings align with previous literature exploring audiologists’ attitudes and practices regarding psychosocial approaches to hearing care, this study is unique in that it is the first sampling of audiologists in reference to care provided to CI patients and their families.

### 4.1 Attitudes toward psychosocial care

Our study found that audiologists working with CI patients and families recognize the value of professional psychosocial intervention. All participants indicated that they believed at least some patients would benefit from referral to a professional specializing in psychosocial care and that intervention would be beneficial. This aligns with prior evidence suggesting audiologists have positive attitudes toward psychosocial intervention in pediatric (23) and adult (39, 44, 47, 48) audiological care. Audiologists recognize their role in psychosocial care (23, 43), and express interest developing knowledge and skills required to appropriately address mental health needs among their patients (44, 48).

### 4.2 Practice patterns related to psychosocial care

Audiology practice patterns related to counseling and psychosocial care are incongruent with audiologists’ positive attitudes toward patient-centered practice. While, audiologists engage in conversations about psychosocial well-being with their patients at some point in their careers, frequencies of this engagement vary. A survey of audiologists by Laird et al. (43) found that about half of all participants reporting they occasionally discuss these topics with patients. In this study, as well as in research by Bennett et al. (40), only a third of participants routinely ask clients about psychosocial well-being. Participants reported conversations regarding psychosocial well-being were more often initiated by patients, suggesting the need for audiologists to be adequately trained in detecting and addressing psychosocial issues (43). Audiologists generally reported mixed attitudes toward discussing psychosocial needs of patients (39), with several barriers to providing adequate support for patients and families. The results of the present study align with previous literature as it reflects low engagement in psychosocial aspects of audiological care. Limited use of mental health screeners and low referral rates underscore the potential benefits of standardized clinical practice in providing patients with appropriate mental health support.

#### 4.2.1 Mental health screeners

Psychosocial screenings are highly effective method to detect clinically significant psychosocial symptoms which warrant counseling with the audiologist or a referral to a counseling professional. Many commonly used psychosocial screeners, such as the GAD-7 (45) and PHQ-9 (46), can be administered in 1–3 min and are found to be suitable for inclusion in audiological practice (49). Screeners are available to specifically measure psychosocial factors in both children and adults with hearing loss, such as the Acceptable and Action Questionnaire-Adult Hearing Loss (AAQ-AHL) and Acceptable and Action Questionnaire-Management of Child Hearing Loss (AAQ-MCHL) (50). Screening for mental health can be valuable in guiding psychosocial intervention among audiologists and is specifically included in the scope of practice for audiologists (25, 37, 38); however, screening tools are underutilized in hearing health care (40).

In our study, no respondents reported routinely administering screenings for psychosocial health, and few participants have ever administered any psychosocial screening, thus putting the burden of reporting mental health concerns onto the patient. These estimates align with previous research regarding psychosocial screening practices among audiologists. Audiologists have described their hesitance in screening for mental health (49) and providing mental health support is due to factors such as lack of confidence and skills, time constrains, and uncertainty about the scope of practice (23, 51, 52). A study by Muñoz et al. (49) found that audiology clinicians reported mixed feelings regarding the administration of mental health screenings, with many audiologists reporting concerns that the patients would feel negatively toward answering screening questions related to mental health, and concerns that they did not have adequate skill or ability to administer the screenings. In contrast, the same study found both adult patients and parents of pediatric patients responded in a highly positive manner to screening regarding depression and anxiety in audiology practice. Specific feedback indicated that the respondents were comfortable with the screeners, and they appreciated the audiologists inquiring about their mental health. Findings from this study are comparable to those of other studies in which audiologists reported not to regularly use mental health screeners or other structured questionnaires as a part of clinical practice (39, 48). The use of mental health screeners would take the burden of initiating concerns related to psychosocial health of the patient and allow audiologists to guide counseling in a way that these concerns are addressed.

#### 4.2.2 Referral indications related to psychosocial care

Audiologists reported a consensus in favor of referring for professional psychosocial care in three situations: suspected self-harm, suspected abuse, and suspected neglect. In many parts of the world, including the United States, audiologists have a legal obligation to report abuse and neglect of vulnerable populations, although mandated reporter laws vary by state (53). Regulations related to reporting self-harm are more nuanced and managed at the state level, and many healthcare settings have strict guidelines for referral in all three situations. These regulations provide audiologists with a clear definition of when a professional specializing in psychosocial care should be engaged as part of interdisciplinary care. A majority of participants also indicated they would make a referral if a patient or family member explicitly asked for a referral for psychosocial care; although three respondents were unsure if they would make a referral, and one respondent indicated they still would not make a referral in this case.

Responses were most mixed regarding more nuanced situations often observed as part of the CI process, although most participants did reply that they would refer in the situation of grief expression related to hearing loss and indecisiveness regarding the decision to pursue CI. It should be noted that participants were given a general statement, and details regarding any specific situation were not provided which may contribute to the number of participants who reported they were unsure of how they would proceed in more nuanced cases. Complex results may be related to a lack of distinction between the psychosocial counseling which lie in an audiologist's scope of practice, and the criteria for therapeutic intervention from a mental health professional. Beck and Kulzer (25) describe the overlapping scope of practice between audiologists and counselors trained in psychotherapy, and further describe the scope of professional counseling. Even given varied responses, more than half the participants reported they would refer for psychosocial services in six out of seven reported scenarios.

#### 4.2.3 Reported standards of practice related to psychosocial health

Despite audiologists’ indication that they would refer to a professional who specializes in psychosocial care in a range of scenarios, estimated referral rates in current practice were very low among participants. Approximately 15% of all participants responded that they never refer for psychosocial services, even among respondents who indicated that they held positive attitudes toward professional psychosocial intervention and were willing to make referrals given a range of clinical situations. The contrast between positive attitudes toward psychosocial care and inconsistent referral patterns aligns with previous research. A study of Australian audiologists by Bennett et al. (44) indicated audiologists’ willingness to refer when presented cases which aligned with DSM-V criteria for psychiatric illness, but few reported referrals as part of routine audiological care. Additional studies support the finding that audiologists are willing to refer patients presenting with psychosocial needs, but did not know who to refer to, when to make a referral, or how to make a referral (39, 53).

Another explanation for the incongruent responses between attitudes and practice patterns may be related to guidance for audiologists in providing psychosocial care. In our study, formal standards to guiding psychosocial care were limited. Few respondents had a written protocol to guide referrals for psychosocial services, and less than a quarter of respondents indicated they had formal resources to provide to patients who they felt needed a referral. Nearly half of all participants reported having no resources, formal or informal, to provide to individuals who they believe would benefit from psychosocial intervention. The lack of resources for addressing patient mental health in hearing care was also documented in Laird et al. (48), where only a quarter of audiologists reported having the resources needed to appropriately address patient mental health needs. These findings suggest audiologists are likely to implement psychosocial care management given appropriate guidance and resources. Specifically, a majority of audiologists were likely to follow a protocol for implementing psychosocial care, apply a specifical psychological framework for addressing mental health, and provide written materials about mental health to patients. Findings support the need for structure on when and how to refer psychosocial care among hearing health professionals (39).

Our study provided the opportunity for open-ended responses to explore other pathways to psychosocial care initiated by audiologists. Approximately a quarter of participants reported they have referred patients in need of psychosocial care to professionals and other individuals other than psychologists and social workers, with the most common response being peer mentors. The use of informal support services, such as community members and peers, is common among individuals with hearing loss and may have a positive impact on device use (7). Referral to the patient's general practitioner was not mentioned, although a person's primary care physician is generally considered an initial step in addressing suspected mental health illness (55).

Two participants reported that psychosocial evaluation and intervention was a required step in their CI candidacy protocol. These participants worked at pediatric hospitals and reported having dedicated psychosocial personnel on their multidisciplinary CI team. Due to the complex aspects in managing the medical, communicative, and psychosocial aspects of cochlear implantation, multidisciplinary care is inherent to CI management. Coordinated, team-based CI management has been documented since the advent of CI (26) and is linked to improved outcomes in CI patients (56, 57). As cochlear implantation is a routine intervention for individuals with moderate-to-profound hearing loss, psychosocial assessment is no longer considered necessary to determine candidacy except in specific cases where psychosocial needs are so great that they take precedent over communication intervention. Rather, psychosocial intervention should be conducted to primarily guide professional CI case management. Routine involvement of mental health professionals on interdisciplinary CI teams could reduce referral barriers and improve patient psychosocial outcomes.

A small number of participants stated beliefs of a limited role of the audiologists in addressing psychosocial health among CI patients and families. Two participants indicated that they believed it was the otolaryngologist's role to assess and refer for psychosocial care, and one participant indicated that it is the Auditory Verbal Therapist's role to manage psychosocial assessment and referrals. Each of these team members are qualified to provide emotional counseling and make referrals for psychosocial care, and the involvement of other professionals should not prevent audiologists from also playing a role in the psychosocial management of patients. A small percentage of audiologists have the persistent belief that counseling is outside the audiologists’ scope of practice (39, 48), although nearly all audiologists in our study reported at least a willingness to refer for psychosocial services.

#### 4.2.4 Barriers to addressing patient psychosocial health

The inconsistences between attitudes, intended actions, and practice patterns indicate barriers in providing psychosocial care among audiologists. The most common barriers related to counseling practices among audiologists included time available in the appointment, and confidence in counseling abilities, and making referrals. More than half of our participants reported a moderate or more impact of time for counseling on practice patterns. In contrast, few participants reported that time had no impact on counseling practice patterns. Time constraints are often cited as a common barrier to providing patient-centered approaches in audiology (58). A majority of audiologist consider having inadequate appointment time to address psychosocial needs as barrier for delivering emotional counseling among audiologists (23, 39, 43, 44, 54, 59) or assessing need for psychosocial referral (60). The lack of time allowed for addressing patients’ psychosocial needs is pervasive in healthcare and documented in speech language pathology (61) among other healthcare fields (62). Additionally, research suggests that patients will continue to initiate conversations regarding psychosocial issues if not adequately addressed (41). Addressing psychosocial needs in patients and their families earlier in the rehabilitation process could have benefits in saving time in later appointments.

The majority of participants in our study reported that their confidence in counseling had an effect on their practice patterns related to psychosocial care, and over half of participants reported that confidence in making a referral also had an impact.

The relationship between confidence and preparedness in psychosocial counseling is reported in literature for both pediatric (23, 63) and adult audiological care (25, 39, 48, 59). Inadequate preparation for psychosocial care can result in audiologists feeling uncertain or uncomfortable in making recommendations regarding psychosocial health (43, 49). Counseling skills are recommended as part of audiological education, and critical skills including active listening, nonverbal communication, silence, and empathy can be taught and evaluated at the graduate level (25). Audiologists who have received graduate-level training in psychosocial care had more confidence in counseling compared to audiologists who lacked training (23). Our data may suggest a trend toward incorporating counseling education in audiological training in the United States, which may be linked to positive attitudes toward psychosocial intervention. In our study, nearly all participants reported having at least one graduate-level course dedicated to counseling. Of those who did not have a graduate-level course in counseling, most had been licensed 12 years or more indicating that further supporting the trend toward graduate-level training in counseling. Even with counseling education offered in audiology training, the availability and quality vary among audiology training programs (24), and educators associated with audiology training programs report ongoing challenges in providing feedback, evaluating student performance, and supporting student learning (24).

In our study, most participants reported little impact of knowing when and where to refer. Audiologists should have the knowledge and skills to recognize the need for psychosocial referral when patient needs expand beyond the audiologists’ scope of practice (25, 48). Previous research indicates that knowing when and how to refer can be a barrier to refer for psychosocial care (39) In addition to a more systematic approach to graduate-level training in counseling skills to clinician education, clarification of the audiologist scope of practice could be valuable in improving patient-centered care among individuals with hearing loss. Thus, the authors support a combination of educational training in the provision of counseling and interdisciplinary team-based practice which includes mental health professionals to best identify and treat the complex psychosocial needs of CI users and their families (64).

#### 4.2.5 Clinical considerations

This study has important clinical implications for audiologists and multidisciplinary CI teams. Individuals experience hearing loss to the degree of qualifying for a CI are at an increased risk for requiring psychosocial intervention when compared to the general population and would benefit from patient-centered focus of addressing psychosocial needs. Members of CI teams should have confidence in assessing and addressing psychosocial needs among patients and families, including the ability to make a referral when psychosocial needs are beyond an audiologists’ scope of practice. This study determined that audiologists working with CI patients and their families hold generally positive attitudes toward the appropriateness and effectiveness of psychosocial intervention, yet practice patterns reflect low involvement of professionals who specialize in psychosocial care. Insufficient clinical time and confidence in abilities related to counseling are barriers to addressing psychosocial needs in this population. Existing research supports improved education of audiologists as a means to increase confidence in detecting and addressing the needs of individuals with hearing loss. Expanding CI teams to include trained professionals such as social workers or psychologists can improve psychosocial care by supporting hearing care professionals when needs extend beyond the audiologists’ scope of practice and providing an established referral pathway for psychosocial intervention. Additionally, interprofessional practice would allow mental health professionals to gain skills in working with CI patients and their families, such as improving communication strategies related to communicating with individuals with hearing loss. Finally, the establishment of protocols and written resources can aid in increasing the likelihood that audiologists will engage in the provision of patient-centered care related to the psychosocial needs of the patient. Therein also lies an opportunity for both ASHA and AAA to clarify and strengthen their descriptions of the audiologists’ scope of practice.

#### 4.2.6 Future research

In order to clarify the scope of practice for audiologists, future research is needed to better understand the psychosocial needs of children and adults with hearing loss, particularly the unique needs experienced by CI patients. A better understanding of these needs would guide research to best address them in a clinical manner. The integration of mental health professionals into rehabilitation protocols has the potential to lead to positive outcomes in this population. An evidence-based CI team protocol which provides clear guidance on detecting and addressing psychosocial needs should be developed, and research should be conducted to evaluate its acceptance and effectiveness in this population.

### 4.3 Strengths and limitations

Cross-sectional surveys pose inherent limitations. First, the sample size was relatively small, making the conclusions somewhat less generalizable. While chi square testing was attempted, cell counts were too small for reliable comparisons. A small sample size is common in CI research as it is a specialized field which serves a relatively small population. Difficulty in achieving large sample sizes is routinely noted in the field of CI research (65). Due to recruitment methods of an online survey, a response rate could not be calculated. Recruitment methods also allowed for self-selection of those who chose to participation which is a form of selection bias. Demographic information collected from the survey indicated a relative balance of responses from most regions in the United States and a range of settings, years of practice, and populations of care. There was an under-sampling of males in this study, which is to be expected given the gender demographics among audiologists.

An additional limitation of this study is the inclusion of audiologists who work in both pediatric and adult populations. Approximately half the participants (*n* = 33, 49%) reported working with both children and adults, but were not given the opportunity to report their attitudes and practice patterns separately in regard to each population. For that reason, responses are generalized and cannot be specified to pediatric or adult care.

Participants were aware that the purpose of the study was to explore audiologists’ attitudes and practice patterns related to psychosocial care, therefore it is likely that audiologists most interested in psychosocial care would engage the survey. This study also has a risk of social response bias in which audiologists may have overstated their attitudes and intended behaviors due to internal pressure to present themselves favorably. The researchers attempted to mitigate this risk by anonymizing the survey. Additionally, reported attitudes cannot be assumed to reflect behavior, presenting an opportunity for future research to measures actual clinical practice patterns among CI audiologists. Further, while the questionnaire was carefully designed by researchers with expertise in CI and epidemiologic survey methods, it is possible that some questions remained unclear. Future research is needed to further understand psychosocial practice patterns among CI audiologists.

## 5 Conclusion

A sampling of audiologists working with CI patients generally hold favorable attitudes related to psychosocial intervention with CI patients and families. Audiologists generally indicated that they would refer to psychosocial services given a range of scenarios. Despite favorable attitudes, audiologists reported that they are not consistently referring to psychosocial services. Under-referral patterns may be related to a lack of formal guidelines for referring patients to psychology or social work. Opportunities exist to advance patient-centered care in CI management by improving graduate-level psychosocial training among audiologists, integrating interprofessional team-based care, and clarifying audiologists’ scope of practice. More research is warranted to explore the disconnect between the recognition of psychosocial needs among CI patients and practice patterns among CI audiologists.

## Data Availability

The raw data supporting the conclusions of this article will be made available by the authors, without undue reservation.
