# Supplementary material for: Audiologists’ attitudes and practice toward referring for psychosocial intervention with cochlear implant patients
Source: Front Rehabil Sci. 2024 Jan 4;4:1306485. doi: 10.3389/fresc.2023.1306485 (PMC10794524; doi:10.3389/fresc.2023.1306485)
Supplement: Supplementary file 1 [file Datasheet1.pdf]

Identifying Approaches Used by Audiologist for Addressing Psychosocial Issue Affecting Cochlear Implant Users

---

Start of Block: Introduction

The University of Memphis Speech and Hearing Center Cochlear Implant Program generated this survey with hopes to better understand audiologist's perspectives on protocols for providing emotional support for hearing-related services, including before or after cochlear implantation. In addition, this study seeks to understand (1) clinical or evidence-based practice related to emotional counseling provided by audiologists and (2) the use of interprofessional support and resource, if any.

For this study, we define counseling as the process of advising an individual, family, or group during one or more sessions to support the operation of overcoming psychosocial issues related to hearing impairment that can include environmental, emotional, or social problems that are affecting the quality of life of a hearing-impaired person.

This survey will take approximately 15-20 minutes to complete. By continuing with this survey, you provide your consent to participate in this study voluntarily. You may choose to stop the survey at any time or leave responses blank if you do not wish to answer. This study has been reviewed by the Institutional Review Board at the University of Memphis, who can be contacted at IRB@memphis.edu. There is no direct potential benefit from participating in this survey. Your identity will be kept confidential. If you have any questions regarding this survey, you can contact Autumn Barron at amlewis8@memphis.edu. The faculty advisor for this study is Sarah Emily Warren, Au.D., Ph.D., MPH, who can be reached at swarren8@memphis.edu. We highly appreciate your participation!

End of Block: Introduction

Start of Block: Demographics

Q1 First, we are interested in your demographics.

Q2 Please indicate what year you first obtained your license to practice audiology.

____

Q3 2. Please select your gender.

- ◯ Male (1)
- ◯ Female (2)
- ◯ Non-binary / third gender (3)

Page Break

Q4 In what region are you currently working?

○ Northeast (1)

○ Southwest (2)

○ West (3)

○ Southeast (4)

○ Midwest (5)

○ Outside of U.S. - Canada (6)

○ Outside U.S. - Europe (7)

○ Outside of U.S. - Other (8)

____

Q5 What is your highest level of degree obtained?

○ M.S./M.A. (1)

○ Au.D. (2)

○ Ph.D. (3)

○ Au.D./Ph.D. (4)

○ Other (5) ____

Page Break

Q6 What setting most accurately reflects your current employment setting?

- Schools (Preschool, K-12) (1)
- University/College Clinic (2)
- School for the Deaf (3)
- Research (Faculty, Private Lab) (4)
- Hospital (5)
- ENT Clinic (6)
- Private Practice (7)
- Industry/Manufacturer (8)
- Other (9) ____

Q7 How many years have you worked as an audiologist?

____

Q8 How many years have you worked as an audiologist specializing in cochlear implants?

____

Q9 What population do you work with?

- ◯ I only work with adult CI patients. (1)
- ◯ I only work with pediatric CI patients. (2)
- ◯ I work with pediatric and adult CI patients. (3)

End of Block: Demographics

Start of Block: Counseling

Q42 Next, we would like to ask questions about your ideas regarding counseling.

Q10 What percentage of adult/pediatric patients (and their families) do you think would benefit from counseling from a psychosocial professional (i.e., social worker, psychologist)?

- ◯ I think all (or nearly all) patients would benefit from professional counseling for psychosocial services. (1)
- ◯ I think most patients would benefit from counseling from a psychosocial professional. (2)
- ◯ I think about half of all patients would benefit from counseling from a psychosocial professional. (3)
- ◯ I think a few patients would benefit from counseling from a psychosocial professional. (4)
- ◯ I don't think any patients would benefit from counseling from a psychosocial professional. (5)

Q11 For the average patient who you would refer for psychosocial counseling, how effective do you think these services would be?

- ◯ Extremely beneficial (1)
- ◯ Very beneficial (2)
- ◯ Probably beneficial (3)
- ◯ Probably not beneficial (4)
- ◯ Not beneficial (5)

End of Block: Counseling

Start of Block: Training and preparedness

Q43 Next, we will ask about your training and preparedness to deliver counseling.

Q12 In your graduate program, how many counseling-specific courses did you take? We define a counseling course that included research on psychosocial and behavioral adjustment to hearing impairment and the impact of cognitive status, general health, and stigma on functional communication and social interaction of adults or children and their families. In addition, the course should explore appropriate counseling skills and strategies in both individual and group settings.

____

Q13 How many counseling-specific hours of CEU training have you had in the past year?

○ 0 - 1.0 hr (1)

○ 1.0 - 2.0 hrs (2)

○ 2.0 - 3.0 hrs (3)

○ 3.0 - 4 hrs (4)

○ > 5.0 hrs (5)

End of Block: Training and preparedness

Start of Block: General Practice

Q44 The next set of questions asks about your general practice with patients, and your clinic's policies.

Q14 Please estimate how much time you spend (in minutes) with an average cochlear implant patient during a typical activation series appointment.

	0 10 20 30 40 50 60 70 80 90 100
Click to write Choice 1 ()	

Q15 Please estimate how much time you spend (in minutes) with an average cochlear implant patient (experienced users) at a typical follow-up appointment.

	0 10 20 30 40 50 60 70 80 90 100
Click to write Choice 1 ()	

Q16 Does your clinic have a written or specific protocol related to emotional support for cochlear implant patients?

- ◯ Yes (1)
- ◯ No (2)

Q16 Please identify the self-assessments/questionnaires you administer to CI patients to aid in counseling addressing psychosocial issues, if any.

	Always (1)	Sometimes (2)	Never (3)
Self-Assessment of Communication (SAC) (1)	○	○	○
Significant Other Assessment of Communication (SOAC) (2)	○	○	○
The Client-Oriented Scale of Improvement (COSI) (3)	○	○	○
Abbreviated Profile of Hearing Aid Benefit (APHAB) (4)	○	○	○
The Glasgow Hearing Aid Benefit Profile (5)	○	○	○
Hearing Handicap Inventory for Adults (HHIA) (6)	○	○	○
Hearing Handicap Inventory for the Elderly (HHIE) (7)	○	○	○
Speech, Spatial, and Qualities of Hearing Questionnaires (SSQ) (8)	○	○	○
Speech, Spatial, and Qualities of Hearing Questionnaires-12 (SSQ12) (9)	○	○	○
Cochlear Implant Function Index (10)	○	○	○
Satisfaction with Amplification in Daily Life (SADL) (11)	○	○	○
International Outcome Inventory	○	○	○

for Hearing Aids (IOI-HA) (12)			
Generalized Anxiety Disorder (GAD-7) Assessment (13)	○	○	○
Patient Health Questionnaire (PHQ-9) (14)	○	○	○
Hearing-Impaired MoCA (HI-MoCA) (15)	○	○	○
Any other questionnaire ____ (16)	○	○	○

Display This Question:

If Please identify the self-assessments/questionnaires you administer to CI patients to aid in couns... = Any other questionnaire ____ [Always]

Q17 You indicated in question 16 that you administer other questionnaires to CI patients to aid in counseling addressing psychosocial issues. Please list these questionnaires you use.

____

Q18 What factors influence your counseling patterns?

	Impacts a lot (1)	Impacts a moderate amount (2)	Impacts a little (3)	Does not impact (4)
My counseling patterns are influenced by the amount of time I have available for counseling. (1)	○	○	○	○
My counseling patterns are influenced my ability to bill for counseling/lack of reimbursement (2)	○	○	○	○
My counseling patterns are influenced by my comfort level with providing counseling (3)	○	○	○	○
My counseling patterns are influenced by my patient's perceived need for counseling. (4)	○	○	○	○
My counseling patterns are influenced by social stigma related to counseling. (5)	○	○	○	○
My counseling patterns are limited because I'm unsure of when to refer. (6)	○	○	○	○

My counseling patterns are limited because I am unsure of who to refer to. (7)	○	○	○	○
My counseling patterns are influenced by my comfort level with referring for counseling. (8)	○	○	○	○

Q19 Would you refer a patient who presented with_______?

	Yes (1)	No (2)	Unsure (3)
patient expresses motivation for self-harm (1)	○	○	○
suspicion of abuse (2)	○	○	○
suspicion of neglect (3)	○	○	○
grief related to hearing loss (4)	○	○	○
patient cries in appointment (5)	○	○	○
patient requests a referral explicitly (6)	○	○	○
patient is not making progress with a device as anticipated (7)	○	○	○
patient has unrealistic expectations (8)	○	○	○
patient is exhibiting signs of cognitive decline (9)	○	○	○
patient has excessive fear that the device will not work or will fail (10)	○	○	○
indecisiveness of next steps (to get an implant or not) (11)	○	○	○

End of Block: General Practice

Start of Block: Practice Patterns

Q47 For the next questions, we are going to ask about your practice patterns for referring to counseling professionals, such as a social worker or psychologist.

Q20 How often do you refer adult/child CI patients to psychology?

- ○ Always (1)
- ○ Most of the time (2)
- ○ About half the time (3)
- ○ Sometimes (4)
- ○ Never (5)

Q21 How often do you refer adult/child CI patients to social work?

- ○ Always (1)
- ○ Most of the time (2)
- ○ About half the time (3)
- ○ Sometimes (4)
- ○ Never (5)

Q22 Does your clinic provide patients with a list of local psychologists, social workers, or other counseling support personnel?

- ◯ Yes, this list is provided to every patient (1)
- ◯ Yes, this list is compiled and is provided to patients as needed (2)
- ◯ We do not have a formal list, but we have some common suggestions (3)
- ◯ No, we do not have a list like this (4)

Q23 Do you refer to any other type of counselor?

- ◯ Yes (1)
- ◯ No (2)

Display This Question:

If Do you refer to any other type of counselor? = Yes

Q24 If so, what kind? For example, this could be a school counselor, religious support person, or a peer mentor.

____

End of Block: Practice Patterns

Start of Block: Block 6

Q53 Finally…

Q25 Are there any other approaches to the psychosocial management of cochlear implant patients that you were not asked about that you would like to describe?

____

End of Block: Block 6
